# Exclusive breastfeeding practice and associated factors among mothers in Motta town, East Gojjam zone, Amhara Regional State, Ethiopia, 2015: a cross-sectional study

**DOI:** 10.1186/s13006-017-0103-3

**Published:** 2017-02-27

**Authors:** Tilahun Tewabe, Alemnesh Mandesh, Tenaw Gualu, Girma Alem, Getnet Mekuria, Haymanot Zeleke

**Affiliations:** 1 Bahir Dar University, College of Medicine and Health science, Bahir Dar, Ethiopia; 2 Addis Ababa University College of Health Sciences School of Allied Health Sciences, Addis Ababa, Ethiopia; 3 DebreMarkos University, College of Medicine and Health science, Bahir Dar, Ethiopia

**Keywords:** Exclusive breastfeeding, Prevalence, Motta, Ethiopia

## Abstract

**Background:**

Exclusive breastfeeding means babies are given only breast milk and nothing else: no other milk, food, drink, not even water for one day (24 hrs) before the survey was conducted. It prevents 13% of childhood mortality; i.e, at least 1.2 million children worldwide would be saved every year. The objective of this study was to assess the prevalence exclusive breastfeeding (EBF) practice and its associated factor among mothers who have infants less than six months of age in Motta town, East Gojjam, Amhara Regional State, Ethiopia.

**Method:**

A community based quantitative cross-sectional study was conducted from April 7, 2015 to May 7, 2015. A simple random sampling technique was applied after taking all registered mothers who have infants less than six months old from local health extension workers of each kebele. A total of 423 mothers with infant less than six months old were included in this study.

The data was collected using an interviewer administered questioaire. Both bivariate and multivariate logistic regression analyses were used to identify factors associated with exclusive breastfeeding practice.

**Result:**

Prevalence of exclusive breastfeeding was 50.1%. Mothers with young infants aged 0-1 month (Adjusted Odds Ratio [AOR] 3.86: (1.64, 9.07), unemployed mothers (AOR 3.01: 1.46, 6.20), low income mothers (AOR 3.61: 1.75, 7.45), mothers who received breastfeeding counseling in pregnancy (AOR 2.76: 1.52, 4.99), fed colostrum (AOR 3.50: 1.45, 8.45), didn't give prelacteal feeds (AOR 4.48: 1.82, 11.03) and were supported by their husband (AOR 2.67: 1.04, 6.95) were more likely to practice exclusive breastfeeding than their counterparts.

**Conclusions:**

Prevalence of exclusive breastfeeding practice in study area was lower than country recommended level. Age of the child, maternal occupation, income, breastfeeding counseling during antenatal care, husband support of breastfeeding and colostrum feeding were independent predictors of exclusive breastfeeding practice. Recommendations to increase exclusive breastfeeding practice are revising postpartum maternity leave, increasing health professional's habit of breastfeeding counseling through training, involving husbands during counseling, educating mothers and the community as a whole to avoid traditional practices that hinder exclusive breastfeeding up to six months.

## Background

Exclusive breastfeeding provides all infants nutritional and fluid needs in the first six months and is a perfect combination of proteins, fats, carbohydrates and fluids [1]. Exclusively breastfeed children are at a much lower risk of infections [2–4] and it is the best and cost effective intervention to reduce infant morbidities and mortalities [1].

Over two-thirds deaths occurring world wide during the first year of life children are often associated with inappropriate feeding practices, especially due to poor exclusive breastfeeding practices [5]. Suboptimal breastfeeding contributes to 45% of neonatal infectious deaths, 30% of diarrheal deaths and 18% of acute respiratory deaths among under five years of age children in developing countries [6]. It also accounts on 10% of the disease burden in children less than 5 years old [7].

A total of 96% of all infant deaths i.e. 1.24 million deaths occur during the first six months of life are attributable to non exclusive breastfeeding which is much higher in Asia and Africa. It accounts 55% of diarrheal deaths and 53% of acute respiratory deaths in the first six months of life [8]. Compared with exclusive breastfeeding in the first few months of life, partial or no breastfeeding is associated with a 2.23-fold higher risk of infant deaths resulting from all causes and 2.40 and 3.94 fold higher risk of deaths attributable to pneumonia and diarrhea, respectively [9].

Non exclusive breastfeeding is known to compromise the nutritional status of children. It results an estimated 40% of under-five stunting in Western and Central Africa (WCA) and more than 60% in some other countries [10].

Exclusive breastfeeding from birth to six months has the potential to prevent 13% of child mortality [2]. However, no more than 35% of infants worldwide are exclusively breastfeeding during the first four months of life [10]. Only 38% of children less than six months of age are exclusively breastfed in the developing countries [11] and 21% in WCA [10].

In Ethiopia suboptimal breastfeeding practices are the major contributor to an estimated 70,000 infant deaths per year, 24% of the total infant death annually and which can be significantly prevented by nutrition interventions such as exclusive breastfeeding [11].

In Ethiopia, 52% of children less than six months old are exclusively breastfed [12]. The Ethiopian Health Sector Development Program (HSDP) IV planned to increase in the proportion of exclusively breastfeeding infants under the age of six months to 70% by the end of 2015 [13].

Breastfeeding and good nutrition for children are recognized as essential for achieving the Millennium Development Goals (MDG), particularly the goals relating to child survival, such as reducing child mortality by 2/3 between 1990 and 2015 [5, 8].

Therefore the purpose of this study was to assess exclusive breastfeeding practices and the associated factors among mothers of children less than six months old in Mottatown, East Gojjam zone, Ethiopia.

## Methods

### Study setting and participants

A community based quantitative cross-sectional study was conducted from April 7 to May 7, 2015.

The study was conducted in Motta town which is located in the Amhara National Regional State, East Gojjam Zone, Ethiopia. It is bordered on all sides by Hulet Ejju Enesse woreda. It is 371 kms away from Addis Ababa. Motta town was founded in 1754. The town has a total of 4 kebeles.The population of the town is 33,500, of which 520 were children less than six months of age. The town has a total of 17 governmental and non governmental health care institutions on work. These are one hospital, one health center, five clinics, one pharmacy and nine drug stores.

The sample size was calculated using single population proportion formula by considering the following assumptions; *p* = 49.1% proportion of exclusive breastfeeding practice which was the prevalence of exclusive breastfeeding in Bahir Dar town, North West Ethiopia [14]. Level of confidence 95%, margin of error (*d*) = 5% and 10% non- response rate. Using a simple random sampling technique 423 mothers participated in the study. To select study participants from each kebele, first the sample size was proportionally allocated to size and finally a lottery method was used to select each study participant. Actual age of the infants was determined by asking the mothers and reviewing the birth date certificate.

### Measurement

A structured interviewer administered questionnaire was used to collect data from participants or mothers of a child. It was constructed by adopting from Ethiopia Demographic and Health Survey (EDHS) 2011 [12] and from previous research done on a similar topic [14, 15] and modified accordingly. A one day (24 hour) infant diet recall method was used for assessing exclusive breastfeeding. Four diploma nurses were recruited as data collectors and two Bachelor of Science nurses were recruited as supervisors. All data collectors and supervisors were oriented and trained on how to interview and record the data for one day before the start of the survey.

### Operational definitions

Exclusive breastfeeding: If an infant was fed only breast milk (with the exception ordered medicines and vitamins by health professionals) one day (24 hrs) before the survey was conducted [12].

Predominant breastfeeding: if an infant mainly took breast milk with non-milk liquid foods such as plain water, tea, salt/sugar solution and juices one day (24 hrs) before the survey was conducted [16].

Mixed breastfeeding: if an infant was fed breast milk with addition of liquid foods like cow milk and formula milk and soft foods like mashed potatoes/meat, porridge, egg, butter one day (24 hrs) before the survey was conducted [16].

Exclusive replacement feeding: If an infant was fed other foods without breast milk one day (24 hrs) before the survey was conducted [12].

### Statistical analysis

The collected data was checked manually for completeness and consistencies, and then it was coded and entered in EPI Info version 3.5.3 and exported to SPSS version 16 for analysis. Descriptive statistics was used to summarize the socio-demographic characteristics’ of the study participants and the prevalence of exclusive breastfeeding.To identify factors associated with exclusive breastfeeding practice, binary logistic regression analysis was carried out at two levels, first bivariate logistic regression was performed to each independent variable with the outcome variable and those variables with a *p* value < 0.05 were included in the final model (multivariate analysis). The strength of association was measured using odds ratio, and 95% confidence intervals. Statistical significance was declared at a *p* value < 0.05.

## Results

### Sociodemographic characteristics

Out of 423 eligible mothers, 405 were participated in this study, which made a response rate of 95.7%. More than half (58.8%) of mothers were below 30 years of age. Most (95.6%) of mothers were Amhara by ethnicity. With regard to educational status, 242 (59.8%) mothers were educated. Around one fifth (20.4%) of study participants were employed mothers. The average household income of the respondents was 1524.26 Ethiopian Birr per month (standard deviation [SD] +1259.65), and 207 (51.1%) respondents earn above 1000 Ethiopian Birr per month (Table 1).

**Table 1:** Socio-demographic characteristics mothers (respondents) who have infants less than six months old, in Motta town, East Gojjam Zone, Ethiopia, 2015

Variable	Category (*n* = 405)	Frequency	Percent (%)
Age of mother( in years)	15-24	111	27.4
	25-29	127	31.4
	30 and above	167	41.2
Religion	Orthodox	229	56.5
	Others^a^	176	43.4
Ethnicity	Amhara	388	95.8
	Others^b^	17	4.2
Level of education of mother	Unducated	163	40.2
	Educated	242	59.8
Occupational status of mother	Employed	82	20.3
	Unemployed	323	79.6
Current marital status	Married	347	85.7
	Unmarried^c^	58	14.3
Husband educational level	Unducated	262	59.5
	Educated	143	40.5
Husband occupation	Employed	111	31.5
	Unemployed	294	68.5
Type of family	Nuclear family	328	80
	Extended family	77	20
Household income (Ethiopain birr)	<1000	198	48.9
	1001-2000	107	26.4
	>2001	100	24.7

^a^Muslim, Protestant and Catholic

^b^Oromo, Tigrie and Gurage

^c^single, widowed and separated

### Infant and maternal health service utilization characteristics

Almost one third (27.4%) children were first in birth order. More than half 231 (57.0%) of infants were between 120-180 days old. A majority (86.2%) of the mothers had an antenatal visit during their pregnancy and 206 (59.0%) were counseled concerning breastfeeding. Mothers who received postnatal care 186 (94.4%) were told about exclusive breastfeeding up to six months of age (Table 2).

**Table 2:** Infant and maternal health service utilization characteristics of study participants in Motta town, East Gojjam zone, Ethiopia, 2015

Variable	Response (*n* = 423)	Frequency	Percent (%)
Sex of infant	Male	180	44.4
	Female	225	55.6
Age of infant	0-60 days	55	13.6
	61-120 days	119	29.4
	121-180 days	231	57.0
Birth order	First	110	27.2
	Second and above	294	73.8
Birth interval	Up to three years	236	58.3
	Three years and above	169	41.7
Antenatal care (*n* = 405)	Yes	349	86.2
Counseling related breastfeeding during antenatal follow up (*n* = 349)	Yes	206	59.0
Place of birth	Health facility	338	83.5
	Home	67	16.5
Mode of delivery	Vaginal /normal	367	90.6
	Caeserian section	38	9.4
Postnatal care (*n* = 405)	Yes	197	48.6
Counseling regarding to breastfeeding during postnatal care (*n* = 197)	Yes	186	94.4

### Exclusive breastfeeding practices

The prevalence of exclusive breastfeeding practice one day before the survey was 50.1% (95% CI: 45.22, 54.98%). Among mothers who didn’t exclusively breastfeed their infant, the main reasons mentioned were; perception of breast milk only being not sufficient for the infant 61 (30.2%), lack of time 46 (22.8%), and decreased breast milk secretion 38 (18.8%) (Table 3)*.*

**Table 3:** Breastfeeding related practices of mothers who have infants less than six months old in Motta town, East Gojjam zone, Ethiopia, 2015

Variables	Responses (*n =* 405)	Frequency	Percent
Colostrum feeding (*n* = 405)	Yes	323	79.8
Infant feeding practice one day before the survey	Exclusively breastfeeding	203	50.1
	Predominantly breastfeeding	156	38.5
	Mixed breastfeeding	46	9.9
	Exclusive replacement feeding	6	1.5
Reasons for not exclusively breastfed (*n* = 202)	Decreased breast milk secretion	38	18.8
	Breast milk only not sufficient for infant	61	30.2
	Infant becomes thirsty	25	12.4
	Lack of time	46	22.8
	Maternal illness	26	12.9
	Breast problem	6	3.0
Who influenced you to give other feeding (*n* = 202)	Husband/spouse	39	19.3
	My mother	23	11.4
	Mother in law	3	1.5
	Health worker	6	3.0
	My own decision	131	64.8

The majority (87.8%) of mothers were supported by their husband to feed their infant exclusively on breast milk. Among employed mothers only 4 (3.7%) were encouraged by their organization/employer to breastfed their infant (Table 4).

**Table 4:** Type of support systemand source of information of mothers about exclusive breastfeeding among those who have an infant less than six monthold in Motta town, East Gojjam zone, Ethiopia, 2015

Variables	Responses	Frequecy	Percent (%)
Husband support (*n* = 353)	Yes	310	87.8
Religious father support (*n* = 405)	Yes	20	4.9
Organizational support of EBF (*n* = 82)	Yes	4	3.7
Cultural support of EBF (*n* = 405)	Yes	2	0.5
Source of information about exclusive breastfeeding	Radio	34	9.4
	Television	174	48.1
	Health extension workers	307	84.8
	Health workers	240	66.3
	Relatives/neighbors/ friends	32	8.8

### Factors associated with exclusive breastfeeding

First variables were tested using bivariate analysis. Variables which were associated (*p* < 0.05) in the bivariate analysis were tested in the final multivariate analysis to see their significant association with exclusive breastfeeding practice.

The independent predictors of exclusive breastfeeding were; age of infant, occupation of mother, marital status, income of the household, antenatal care, breastfeeding counseling during antenatal care, place of birth, mode of delivery, postnatal care, timely initiation of breastfeeding, colostrum feeding , prelacteal feeding and husbands support.

The final predictors for exlusive breastfeeding practice were; age of infant, maternal occupation, household income, breastfeeding counseling during antenatal care, husband's support of breastfeeding, colostrum feeding and prelacteal feeding.

An infant who is 0-1 month old was four times more likely to exclusively breastfed than infant aged 4-5 months (AOR 3.86: 1.64, 9.07). Similarly infants aged 2-3 months were almost two times more likely to feed exclusively on breast milk than 4-5 month old infants (AOR 2.24: 1.16, 4.31).

Concerning to occupation, unemployed mothers were three times more likely to practice EBF than employed mothers (AOR 3.01: 1.46, 6.20). Income was significantly associated with EBF practice. Mothers who earn less money (<1000 Ethiopian birr/month) were 3.6 times more likely to practice exclusive breastfeeding than mothers whose average monthly household income was 2001 Ethiopian birr and above (AOR 3.61: 1.75, 7.45). While mothers who earn between 1001- 2000 Ethiopian birr/month were almost two times more likely to practice EBF than those who earn >2001 Ethiopian birr/month (AOR 2.34: 1.12, 4.91).

Mothers who were counseled regarding to breastfeeding during antenatal care were almost three times more likely to practice exclusive breastfeeding than mothers who were not counseled (AOR 2.76: 1.52, 4.99).

Mothers who fed colostrum to their infant were 3.5 times higher to practice EBF than those who didn't feed colostrum (AOR 3.50: 1.45, 8.45). On the other hand, mothers who didn't give prelacteal food to their infant were almost four times more likely to practice EBF than mothers who fed prelacteal food (AOR 4.48: 1.82, 11.03).

Pertaining to support, mothers who had their husband's support for breastfeeding were almost three times more likely to practice EBF than mothers who were not supported by their husband (AOR 2.686: 1.037, 6.953) (Table 5).

**Table 5:** Factors that affect EBF practice among mothers of infants age less than 6 months using bivariate and multivariate logistic regression analysis model, East Gojjam, Ethiopia, 2015

Variables	EBF Practice				
		Yes (*n* & %)	No (*n* & %)	COR (95% CL)	AOR (95% CL)
Age of child in months	0-1	37 (67.3)	18 (32.7)	2.84 (1.53,5.28)	3.86 (1.64, 9.07)*
	2-3	69 (58.0)	50 (42.0)	1.91 (1.22, 2.98)	2.24 (1.16, 4.31)*
	4-5	97 (42.0)	134 (58.0)	1	1
Occupational status	Unemployed	173 (53.6)	150 (46.4)	1.99 (1.21, 3.30)	3.01 (1.46, 6.20)*
	Employed	30 (36.6)	52 (63.4)	1	1
Marital status	Married	182 (52.4)	165 (47.6)	1.94 (1.10, 3.46)	
	Unmarried	21 (36.2)	37 (63.6)	1	
Income	<1000	114 (57.6)	84 (42.4)	2.63 (1.59, 4.35)	3.61 (1.75, 7.45)*
	1001-2000	55 (51.4)	52 (48.6)	2.05 (1.17, 3.59)	2.34 (1.12, 4.92)*
	>2001	34 (34.0)	66 (66.0)	1	1
Antenatal care	Yes	185 (53)	164 (47)	2.38 (1.31, 4.34)	
	No	18 (32.1)	38 (67.9)	1	
Breastfeeding counseling during antenatal care	Yes	131 (63.6)	75 (36.4)	2.88 (1.85, 4.48)	2.76 (1.52,4.99)*
	No	54 (37.8)	89 (62.2)	1	1
Place of birth	Health facility	185 (54.7)	153 (45.3)	3.29 (1.84, 5.89)	
	Home	18 (26.9)	49 (73.1)	1	
Mode of delivery	Normal/vaginal	190 (51.8)	177 (48.2)	2.06 (1.02, 4.16)	
	Ceaserian section	13 (34.2)	25 (65.8)	1	
Postnatal care	Yes	116 (58.9)	81 (41.1)	1.99 (1.34,2.96)	
	No	87 (41.8)	121 (58.2)	1	
Colostrum feeding	Yes	185 (57.3)	138 (42.7)	4.77 (2.70, 8.41)	3.50 (1.45, 8.45)*
	No	18 (22)	64 (78)	1	1
Prelacteal feeding	No	189 (58.7)	133 (41.3)	6.90 (3.73,12.79)	4.48(1.82, 11.03)*
	Yes	14 (17.1)	68 (82.9)	1	1
Husbands support	Yes	174 (56.1)	136 (43.9)	4.22 (2.01, 8.87)	2.69 (1.04, 6.95)*
	No	10 (23.3)	33 (76.7)	1	1

1 = reference, gg * = *p*-value less than 0.05, *n* = number, % = percent

## Discussion

In spite of what is known about the benefit of exclusive breastfeeding; the practice is not satisfactory in the study area. Only half, 50.1% (95% CI: 45.22% - 54.98%) of mothers reported they were exclusively breastfeeding their infant, which is lower than Ethiopian HSDP IV target level of 70% [13]. This result is comparable to the 2011 EDHS 52% [12], EDHS 2005 49% [17] and with other similar studies done in; Bahir Dar, Ethiopia 49.1% [14], Kumasi Metropolis, Ghana 48% [18], Mecha district, NorthWest, Ethiopia 47.13% [19] and Dare Salaam, Tanzania 46% [20]. This finding is higher than; worldwide prevalence 35% [5], SSA 31% [6] and from other studies from; Malaysia 44.3% [15], Injibara, Awi zone 44% [21], Ambo 42.3% [22], Axum 40.9% [23], Bangladish 36% [24], Nairobi, Kenya 34% [25], Egypt 29.9% [26], Sudan 29.5% [27] and Nigeria 20% [28]. However the result is lower than studies from Kigoma, Tanzania 58% [29] and Debre Markos, Ethiopia 60.8% [16]. The difference might be due to methodological variations between studies and differences in sociocultural, economical, health and health service utilization characteristics between respondents of the referenced areas and the study place.

In this study unemployed mothers practiced EBF better than employed mothers. This result is similar to studies from: Malaysia [15, 30], the Netherlands [31], Utah State [32], Cameroon [33], Timor-Leste [34], Ghana [18], Awi Zone, Ethiopia [22], and Debre Markos, Ethiopia [16]. On the other hand mothers with better economical status practised EBF poorly. This is in line with research findings in Cape Metropole [35] and Nigeria. This might be because mothers who earn less money have no any option to buy other food and employed mothers tend not to breastfeed their infant exclusively due to: short period of maternity leave, lack of time, distance of the workplace from home, lack private space for breastfeeding or expressing milk at the workplace, inflexible work schedule, absence of on site or near site child care centers.

In this study breastfeeding counseling during preganancy were found to facilitate exclusive breastfeeding practice. This finding is parallel with studies done with low income Latinos in US [36], Nigeria and Debre Markos, Ethiopia [16]. This could be counseling enhances mothers’ understanding and appreciation of the demands and benefits of EBF. Mothers who were counseled during pregnancy prepared themselves psychologically as well economically to exclusively breastfeed the infant.

Colostrum feeding facilitates exclusive breastfeeding since it promotes the early initiation of breastfeeding which also increases child survival. This finding is consistent with findings in Axum [23] and Dabat, Gonder [37]. Whereas giving prelacteal feeding hinders the practice of EBF, which is similar with findings in Al Hasa, Saudi Arabia [38] and Debre Markos, Ethiopia [16]. This might be due to the fact that when mothers introduce other food to the newborn before breastfeeding, it decreases the infants suckling activity and which in turn affects or decreases maternal milk secretion.

Husbands support had a significant relationship with EBF practice in the study area. A mother who was supported by their husband's practiced EBF better than those who were not supported. Which is similar with findings in Malaysia [30] and Injibara town, Awi Zone, Ethiopia [21]. This might be because in Ethiopia a husband plays a major role in the decision making about family and household issues and which affects many aspects of family life including infant feeding practices.

A limitation of this study was that it assessed only the quantitative aspects of exclusive breastfeeding and it may have underestimated the prevalence of exclusive breastfeeding in the study area due to recall bias and because strength representativeness was increased, since it was a community based study.

## Conclusion

The prevalence of exclusive breastfeeding in the study area was lower than the country recommended level. Age of the child, maternal occupation, income, breastfeeding counseling during antenatal care, husbands support, colostrum feeding and not giving prelacteal feeds were the determinant factors for higher chance of EBF practice in the study area.

Recommendations for improving EBF include; extending maternal leave, constructing on-site or near site child care centers, behavior change communication to avoid traditional activities training of health professionals regarding to infant feeding practices and higher socio-economic class mothers should be given priority. Every arrangement should be made to increase their exclusive breastfeeding practice, community based breastfeeding education and counseling to pregnant women and husbands should be given.

## Abbreviations

ANC: Antenatal Care
AOR: Adjusted Odds Ratio
CI: Confidence Interval
EDHS: Ethiopian Demographic Health Survey
SD: Standard Deviation
UNICEF: United Nation Children Emergeny Fund
WHO: World Health Organization
